# Transpedal approach for femoral-popliteal chronic total occlusions using the outback® elite re-entry device

**DOI:** 10.1186/s42155-020-00203-5

**Published:** 2021-01-06

**Authors:** Mike Gorenchtein, Naveed Rajper, Philip Green, Pankaj Khullar, Daniel Amoruso, Christian Franz Bulacan, Tak Kwan, Joseph Puma, Justin Ratcliffe

**Affiliations:** 1 Department of Cardiovascular Medicine, Mount Sinai Morningside Hospital, Icahn School of Medicine at Mount Sinai, New York, NY USA; 2 Department of Cardiology, Mount Sinai Beth Israel Hospital, Icahn School of Medicine at Mount Sinai, New York, NY USA

**Keywords:** Chronic total occlusions, Femoral-popliteal artery, Alternate access, Transpedal access, Peripheral arterial disease, Endovascular therapy

## Abstract

**Background:**

Transpedal access is increasingly utilized for the treatment of peripheral artery disease (PAD). Femoral-popliteal artery chronic total occlusions (CTOs) are some of the most difficult lesion subsets that sometimes require the use of re-entry support devices during percutaneous intervention. Limited data is available on the use of re-entry devices when treating femoral-popliteal CTOs via transpedal access. The aim of this study was to demonstrate the feasibility of using the Outback® Elite re-entry device for the treatment of femoral-popliteal CTOs via the transpedal approach in an outpatient based lab setting.

**Methods:**

Seventeen patients presented with femoral-popliteal CTOs in which treatment required the use of the Outback® Elite re-entry device. All procedures were performed in a single outpatient based lab. Patients were followed at 1 week and 1 month post-procedure, with lower extremity arterial duplex ultrasound assessment during the 1 month follow-up.

**Results:**

The average patient age was 78 years-old, with 71% being males. Most patients presented with Rutherford class IV symptoms. Procedural success was achieved in all patients with no requirement to convert to femoral artery access in any of the cases. No immediate post-procedural complications nor at any time during follow-up were observed. Ultrasonography at 1 month follow-up showed patent intervention sites and access site vessels in all patients.

**Conclusion:**

The use of the Outback® Elite re-entry device for the treatment of femoral-popliteal CTOs via transpedal access is a feasible option and may have potential benefits by avoiding risks associated with traditional femoral artery access.

## Background

Peripheral arterial disease (PAD) is a common and debilitating condition that affects an estimated 202 million people worldwide (Gerald et al. 2013). More importantly, there has been a greater than 20% increase in the incidence of PAD since 2001(Pande et al. 2011; Gerald et al. 2013). Due to the rapid development of new technologies and techniques, an endovascular approach is a now a first-line option after conservative therapy for the treatment of symptomatic PAD. Between 2001 and 2007, PAD treatment has seen a 78% increase in endovascular procedures and 20% decrease in open bypass procedures with an overall reduction in the need for amputation during that same time period (Hong et al. 2011).

The traditional access site for the endovascular treatment of PAD is the contralateral common femoral artery (CFA) and subsequent cross-over, or ipsilateral antegrade CFA access. However, femoral access site complication rates have been reported to be as high as 2.3% and comprise a significant proportion of overall procedural related complications (Jolly et al. 2009; Sajnani and Bogart 2013). In order to decrease these complication rates, alternative access sites, including pedal and radial artery (RA) access, have become more frequently utilized. Alternative access sites have the benefit of being safer as well as providing increased patient comfort/satisfaction and shorter recovery time/time to ambulation (Kiemeneij et al. 1997; Vora and Rao 2014; Kwan et al. 2015).

A frequent concern that many peripheral operators have with the transpedal approach is the perceived lack of availability of certain devices for use in challenging cases. Chronic total occlusions (CTOs) in the superficial femoral artery (SFA) or popliteal artery remain one of the hardest lesion subsets to treat and sometimes require re-entry from a dissection plane back into the vessel true lumen. In this case series, we show the feasibility of using the Outback® Elite re-entry device for the treatment of femoral-popliteal CTOs via transpedal access.

## Methods

### Patients

Seventeen patients who failed conservative management for lifestyle limiting claudication or critical limb ischemia (Rutherford class III or higher) were identified and underwent diagnostic angiograms which demonstrated femoral-popliteal CTOs. Following discussion of possible treatment options, including vascular bypass, a collective decision was made to proceed with percutaneous endovascular intervention for each patient. All procedures were performed via transpedal access in a single outpatient based lab setting. The transpedal approach was selected due to known prior difficulty and failed standard CTO revascularization attempts in each of the patients.

### Vascular access and lesion identification

All patients were brought to the catheterization laboratory and prepared in standard fashion for a diagnostic unilateral peripheral angiogram. An ultrasound was performed to determine which vessel – anterior tibial artery (ATA), posterior tibial artery (PTA), or peroneal artery would be the appropriate access site. The pedal area on the ipsilateral side of the affected leg was then prepared in sterile fashion and a 21-gauge micropuncture needle was used for arterial access under ultrasound guidance followed by placement of a 4-Fr Glidesheath (Terumo). After confirming arterial flow, 5000 units of heparin were given intravenously and an antispasmodic cocktail of nitroglycerin 100 μg and verapamil 2.5 mg was administered into the sheath. To achieve an activated clotting time (ACT) > 300 s, additional heparin was given if needed. A routine diagnostic angiogram was performed and a femoral-popliteal CTO was encountered in each case. Secondary arterial access was obtained via the RA for antegrade injections. All patients received conscious sedation (fentanyl and/or midazolam) as well as local anesthesia.

### Endovascular intervention and follow-up

The Outback® Elite re-entry device (Cordis) was used after initial wire escalation attempts failed to re-enter into the true lumen of the stenotic lesion. For each procedure, radial access was obtained using a 4-Fr sheath, which was eventually upsized to a 6-Fr Slender Glidesheath (Terumo) to allow use of the Outback re-entry device. Final treatment of the CTO with angioplasty and/or stenting was performed at the discretion of the operator. Procedural success was defined as successful re-entry into the true vessel lumen with < 20% residual stenosis at the target lesion by the end of the procedure. Based on our prior success and low complication rates using the TR band (Terumo) and VasoStat (Forge Medical, Inc.) devices to achieve hemostasis at different transpedal puncture sites, we implemented the same previously described technique in all the cases (Patel et al. 2016). Each patient was discharged home 2 h post-procedure on atorvastatin 80 mg daily and dual antiplatelet therapy with daily aspirin 81 mg and clopidogrel 75 mg for at least 1 month. All patients had clinical follow-up at 1 week and 1 month post-procedure, with lower extremity arterial duplex ultrasound assessment at the 1 month follow-up. Below are 3 selected cases to illustrate how the Outback® Elite re-entry device was utilized in each endovascular intervention.

#### Case #1

**Fig. 1:**
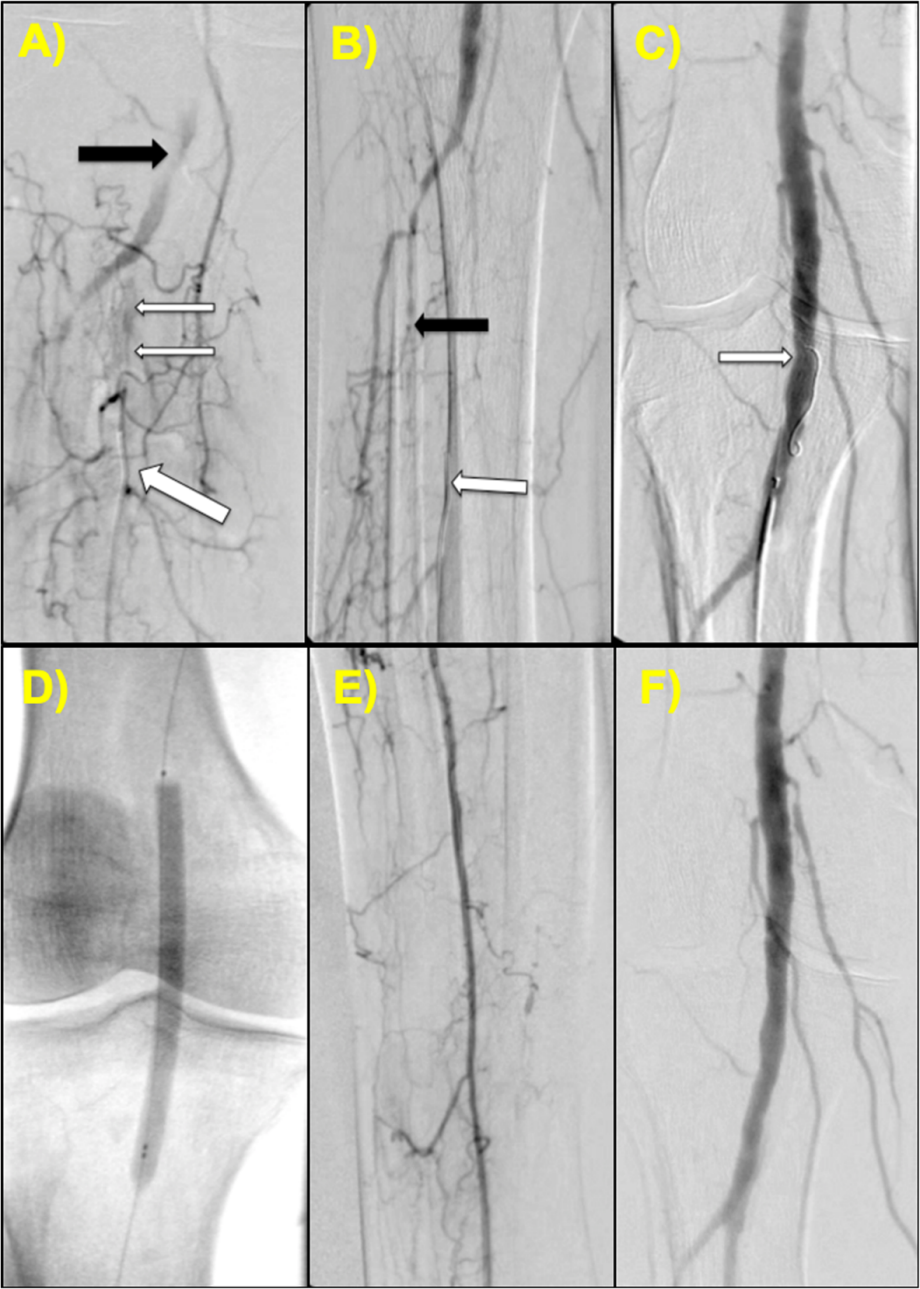
Case #1 (**a**) Retrograde angiography via the right peroneal artery (thick white arrow) demonstrating the tibioperoneal (TP) trunk into the proximal peroneal artery CTO (thin white arrows) with an extensive network of collaterals and vague reconstitution of the native popliteal artery through retrograde filling (black arrow). **b** Antegrade angiogram re-demonstrating the CTO with zero vessel runoff and an occluded proximal ATA (black arrow). Note again the retrograde catheter in the peroneal artery (white arrow). **c** The Outback re-entry device entering the true lumen of the distal popliteal artery (white arrow). **d** Balloon angioplasty via pedal artery access. **e** Post-intervention angiogram showing successful recanalization through the TP trunk and peroneal artery. **f** Post-intervention angiogram of the peroneal artery access site illustrating excellent flow without vessel injury

A 74 year-old male who presented with Rutherford class IV ischemic rest pain was brought to the catheterization laboratory and a right peroneal artery access was obtained. A tibioperoneal (TP) trunk into the proximal peroneal artery CTO with zero vessel runoff was identified (ATA and PTA were also occluded – Fig. 1a). To visualize the full length of the CTO and facilitate re-entry, radial access was obtained with a 4-Fr sheath, followed by advancement of a 4-Fr Multicurve 150 cm catheter (Terumo) into the CFA (Fig. 1b). The 4-Fr pedal sheath was upsized to a 6-Fr Slender Glidesheath (Terumo) to allow use of the Outback re-entry device. Using test injections from the antegrade catheter via radial access, the Outback re-entry device was positioned and advanced over a hydrophobic 0.014-in. Grand Slam wire (ASAHI) with successful penetration into the true lumen of the popliteal artery (Fig. 1c). Angioplasty of the distal popliteal into the TP trunk/peroneal artery was performed (Fig. 1d). Post-angioplasty angiogram showed excellent flow with no dissection – therefore stenting was not necessary (Fig. 1e). Final angiogram of the distal peroneal artery did not reveal any vessel injury (Fig. 1f).

#### Case #2

**Fig. 2:**
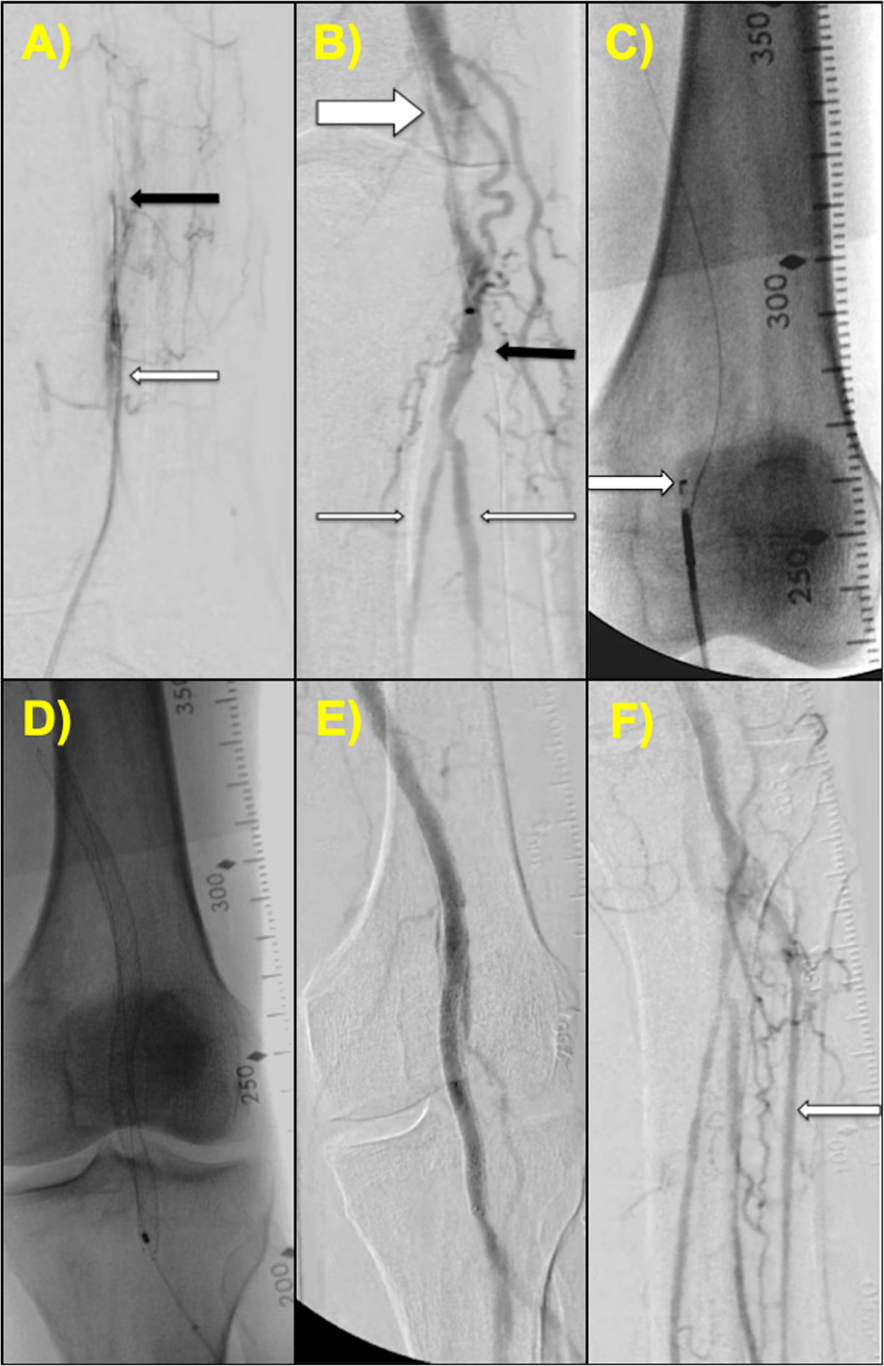
Case # 2 (**a**) Angiogram through the pedal access sheath in the ATA (white arrow) shows a distal CTO of the ATA (black arrow). **b** Antegrade angiogram showing a popliteal CTO. Note the dissection plane created by wire escalation techniques adjacent to the true lumen of the popliteal artery (thick white arrow). The ATA is completely occluded from proximal to distal (black arrow). Collaterals fill the TP trunk into the PTA and peroneal artery (thin white arrows). **c** The Outback re-entry device entering the true lumen of the distal popliteal artery (white arrow). **d** Balloon angioplasty followed by stenting of the popliteal artery. **e** Post-intervention angiogram showing restoration of flow post-stenting. **f** Final angiogram of the infrapopliteal vessels demonstrating direct line flow to all pedal arteries, including the ATA access site (white arrow)

A 79 year-old male was sent for evaluation for worsening claudication that had progressed to Rutherford class IV ischemic rest pain in his left lower extremity. Left ATA access was obtained and angiography revealed a distal CTO of the ATA (Fig. 2a). Left radial access was obtained and a catheter was advanced into the CFA. Antegrade angiogram revealed a popliteal artery CTO. Collateral filling of the PTA and peroneal artery were noted. With wire escalation techniques, a dissection plane was created from the ATA adjacent to the true lumen of the popliteal artery (Fig. 2b). Small balloon inflation using a 3.0 mm × 250 mm Saber balloon (Cordis) of the ATA was performed as part of preparing the vessel lumen for the Outback re-entry device. Using test injections from the antegrade catheter via radial access, the Outback re-entry device was positioned and advanced over a hydrophobic 0.014-in. Grand Slam wire (ASAHI) with successful re-entry into the true lumen of the target vessel (Fig. 2c). Angioplasty and stenting were performed (Fig. 2d) with good post-intervention results (Fig. 2e). Direct 3-vessel outflow was seen at the end of the procedure (Fig. 2f).

#### Case #3

**Fig. 3:**
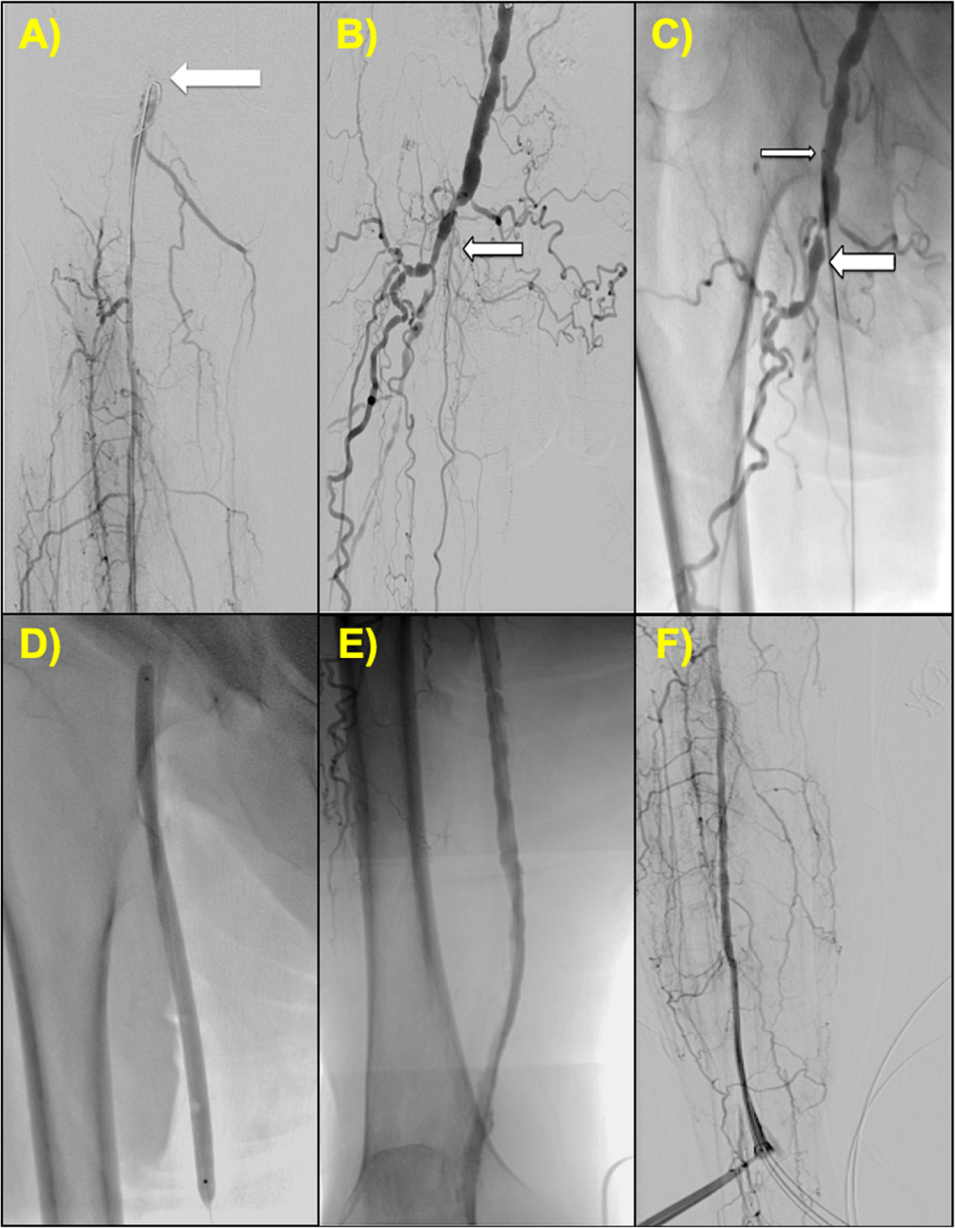
Case #3 (**a**) Retrograde angiography via peroneal access demonstrating the distal cap of the CTO at the popliteal artery (white arrow). **b** Antegrade angiography depicting the proximal cap of the CTO at the ostial SFA (white arrow). **c** The Outback re-entry device traversing the SFA (thick white arrow) with the tip positioned in the CFA for re-entry into the true lumen (thin white arrow). **d** Balloon angioplasty of the occluded SFA. **e** Post-intervention angiogram showing a patent vessel with no significant stenosis. **f** Final angiography of the peroneal access site exhibiting vessel patency and no evidence of vessel injury

A 74 year-old female presented with Rutherford Grade V symptoms - ischemic rest pain and dry gangrene on the plantar surfaces of her right 1st, 3rd, and 5th toes with duplex ultrasonography showing a long CTO of the entire right SFA and extending into the popliteal artery. Right peroneal artery access was obtained and retrograde angiogram demonstrated the distal cap of the CTO at the popliteal artery as well as total occlusion of the ATA and PTA (Fig. 3a). Left radial access was obtained and a catheter was advanced into the CFA. Antegrade injections localized the proximal cap of the CTO to the ostial SFA (Fig. 3b). The Outback re-entry device was advanced over a 0.014-in. Grand Slam wire (ASAHI) and positioned using antegrade injections with successful entry into the true lumen (Fig. 3c). Angioplasty of the entire SFA and popliteal was performed using 6.0 mm × 250 mm Saber balloons (Cordis; Fig. 3d). Post-intervention angiography of the SFA demonstrated patency of the previously occluded vessel (Fig. 3e) and patency of the peroneal artery access site (Fig. 3f).

## Results

The patient and lesion characteristics are listed in Tables 1 and 2. The average patient age was 78 years-old with 71% male. All patients had multiple co-morbidities predisposing to PAD. Most patients presented with Rutherford class IV symptoms. Post-angioplasty stenting was performed in 1/17 patients. Procedural success was achieved in all patients without the need to convert to femoral artery access in any of the cases. The average duration of the procedures was 56 min. No complications immediately post procedure nor at any time period during follow up were noted. All patients reported clinical improvement in symptoms. Duplex ultrasound at 1 month follow-up showed patent intervention sites as well as patent pedal access sites.

**Table 1:** Patient baseline characteristics

Patient	Age	Sex	HTN	HLD	DM	CAD	Smoking	Rutherford class
1	85	M	Yes	Yes	No	No	No	IV
2	78	M	Yes	Yes	Yes	Yes	No	IV
3	78	M	Yes	Yes	Yes	No	Yes	IV
4	82	F	Yes	Yes	No	Yes	Yes	IV
5	80	F	Yes	Yes	Yes	Yes	Yes	IV
6	77	M	Yes	Yes	Yes	Yes	Yes	IV
7	86	M	Yes	Yes	No	Yes	No	IV
8	88	M	Yes	Yes	No	Yes	Yes	IV
9	76	M	Yes	Yes	No	Yes	Yes	III
10	69	M	Yes	Yes	Yes	Yes	No	IV
11	76	F	Yes	Yes	No	Yes	No	IV
12	53	F	No	Yes	No	Yes	No	IV
13	87	M	Yes	Yes	No	Yes	No	IV
14	77	M	Yes	Yes	No	Yes	Yes	III
15	86	M	Yes	Yes	Yes	Yes	Yes	IV
16	69	M	Yes	Yes	Yes	Yes	Yes	III
17	74	F	Yes	Yes	No	No	Yes	V

*M* male, *F* female, *HTN* hypertension, *HLD* hyperlipidemia, *DM* diabetes, *CAD* coronary artery disease

**Table 2:** Procedural characteristics

Patient	Access site	Additional access site	Intervention site	Contrast volume (ml)	Stent used
1	Peroneal artery (L)	RA (L)	TP trunk (L)	80	No
2	ATA (L)	RA (L)	SFA (L)	35	Yes
3	Peroneal artery (R)	RA (L)	POP (R)	55	No
4	PTA (L)	RA (L)	SFA (L)	60	No
5	ATA (R)	RA (L)	SFA-POP (R)	65	No
6	ATA (L)	RA (L)	SFA (L)	35	No
7	ATA (L)	RA (L)	SFA (L)	45	No
8	ATA (R)	RA (L)	CFA (R)	40	No
9	Peroneal artery (L)	RA (R)	SFA (L)	45	No
10	ATA (R)	RA (R)	SFA-POP (R)	40	No
11	ATA (R)	RA (L)	SFA-POP (R)	70	No
12	PTA (R)	RA (L)	SFA-POP (R)	50	No
13	Peroneal artery (R)	RA (L)	POP (R)	60	No
14	Peroneal artery (R)	RA (L)	SFA-POP (R)	50	No
15	ATA (L)	RA (L)	POP (L)	35	No
16	ATA (L)	RA (L)	SFA (L)	35	No
17	Peroneal artery (R)	RA (L)	SFA-POP (R)	40	No

*ATA* anterior tibial artery, *L* left, *POP* popliteal artery, *PTA* posterior tibial artery, *RA* radial artery, *R* right, *SFA* superficial femoral artery

## Discussion

Several approaches are being utilized for the endovascular treatment of femoral-popliteal CTOs. An antegrade intraluminal approach had been the traditional method of revascularization, however, this technique may fail in up to 25% of cases, particularly with long, heavily calcified lesions (Jacobs et al. 2006; Conrad et al. 2006). Intentional subintimal dissection with true lumen re-entry, as first described by Bolia et al., is an appealing alternative strategy for treatment of CTOs, but it is not uncommon to fail true lumen access using a wire alone (Bolia et al. 1990). The Outback re-entry device is one of several support devices that has shown to be successful in transfemoral access, and more recently via transpedal access, when wire escalation techniques fail (Beschorner et al. 2009; Gandini et al. 2013; Patrone and Stehno 2019; Hayakawa et al. 2020). At our own practice, while the majority of femoral-popliteal CTOs can be crossed using standard techniques (mainly wire escalation therapies via transepdal or even dual access), < 5% of cases require the use of a support device. In this case series, we show that the Outback re-entry device can just as easily be utilized in high complexity lesion subsets such as femoral-popliteal CTOs, when wire escalation/dissection techniques fail. While the treatment of complex PAD via a transpedal approach is not new, the feasibility of using re-entry devices for femoral-popliteal CTOs through pedal access sites is not yet widely reported (Scott et al. 2007; Clark et al. 2016). The ability to treat complex PAD via the transpedal approach with the same armamentarium available with the femoral access site is important to realize because one of the concerns that many operators have in adopting the transpedal approach is the availability of support equipment for complex cases.

The endovascular treatment of PAD via the transpedal approach is also a possible way to follow the current trend in healthcare of providing patients with safe, effective care while decreasing costs and minimizing risks. The risks associated with femoral artery access have been well published which has led to the development of smaller, alternative access sites. In our coronary counterparts, radial access has been proven to be a safer alternative to femoral access (Kiemeneij et al. 1997; Jolly et al. 2009; Sajnani and Bogart 2013; Vora and Rao 2014). Other benefits to the transpedal approach may include less contrast utilization, less radiation exposure, and less post procedure monitoring (decreased time to ambulation). This is especially important as many of these procedures can be performed in the outpatient based lab setting – which may decrease health care costs by reducing inpatient hospital monitoring.

Ideally, transpedal retrograde revascularization using the Outback re-entry device is best suited if prior antegrade attempts are unsuccessful and in cases with at least 2 tibial vessels (should access vessel closure occur). Pedal vessel selection and determining the appropriateness of the access site should be guided by duplex ultrasound. In addition, it is important to be aware of potential procedure complications such as vessel perforation, and dissection of the treated or accessed vessel. For these reasons, the contralateral femoral site should be always prepared in case of bailout.

### Limitations

This is a small, single center retrospective series of selected patients and larger studies are necessary to better evaluate safety and efficacy. For the purposes of this paper, follow-up was limited to 1 month and our results cannot be extrapolated to determine long-term vessel patency using this approach. Furthermore, the procedures were performed by operators familiar with transpedal access and the results may not be generalizable to those with less experience in the transpedal technique especially since ultrasound guided access may be difficult and time-consuming to operators unfamiliar with the approach.

While radial access was still necessary to visualize the proximal cap and length of the CTO, smaller 4-Fr sheaths are still utilized and the femoral artery is still avoided which will result in less complications. Furthermore, Clark et al. reported a case of transpedal only access for treatment of a superficial femoral artery CTO - which highlights the fact that in some cases the filling of the proximal cap through collaterals may be sufficient (therefore making radial access unnecessary) (Clark et al. 2016). Although more studies need to be performed to further evaluate the scope of PAD treatment via the transpedal access site, the ability to use re-entry devices via transpedal access is an important step towards increased utilization of alternative access sites.

## Conclusion

The treatment of femoral-popliteal CTOs can be successfully performed using the Outback® Elite Re-entry device via the transpedal approach – an innovative access site that may help with the current healthcare trend of cost containment while increasing patient safety.

## Data Availability

All data generated or analyzed during this study are included in this published article.

## Abbreviations

ACT: Activated clotting time
ATA: Anterior tibial artery
CFA: Common femoral artery
CTO: Chronic total occlusion
PDA: Peripheral arterial disease
PTA: Posterior tibial artery
RA: Radial artery
SFA: Superficial femoral artery
TP: Tibioperoneal
